# Intranasal delivery of mRNA expressing newly identified *Acinetobacter baumannii* antigens protects against bacterial lung disease

**DOI:** 10.1038/s41541-025-01202-0

**Published:** 2025-07-04

**Authors:** Sophie L. Higham, Ziyin Wang, Valarmathy Murugaiah, Jihye Song, Chubicka Thomas, Houze Zhang, Uta Griesenbach, Eric W. F. W. Alton, Luke A. Granger, Andrea Flores Esparza, Beatriz Dias Barbieri, Paul G. Hitchen, Paul Kellam, Robin J. Shattock, Shiranee Sriskandan, Stephen T. Reece, John S. Tregoning

**Affiliations:** 1 Imperial College London, Department of Infectious Disease, London, UK; 2 Imperial College London, National Heart and Lung Institute, London, UK; 3 Imperial College London, Department of Life Sciences, London, UK; 4 RQ Biotechnology Ltd, London, UK; 5 Kymab, a Sanofi Company, Babraham Research Campus, Cambridge, United Kingdom; 6 Present Address: Coalition for Epidemic Preparedness Innovations, Oslo, Norway

**Keywords:** Adjuvants, RNA vaccines

## Abstract

Vaccines are central to the strategy to control antimicrobial resistant (AMR) bacterial infections; one multidrug resistant pathogen of particular concern is *Acinetobacter baumannii*. In this study we identified two novel *A. baumannii* antigens using mass spectrometry and phage expression: Oxa23 and PAL. These genes are highly conserved between different isolates of *A. baumannii* and recognised by convalescent human sera. We explored their protective immunity using two different vaccine platforms, recombinant outer membrane vesicles (rOMV) and mRNA. RNA vaccine immunised mice had significantly reduced bacterial load in their lower airways following challenge with carbapenem resistant *A. baumannii*, with Oxa23 providing better protection than PAL. We then compared routes of delivery and RNA vaccine platforms, demonstrating that intranasally delivery of mRNA encoding OXA-23 (formulated with GL67A) significantly reduced disease severity and enhanced bacterial clearance. These studies validate in silico identified antigens through challenge studies and novel mucosal vaccine delivery approaches.

## Introduction

*Acinetobacter baumannii* is a highly antibiotic resistant Gram-negative bacterium responsible for causing a range of nosocomial infections and hospital outbreaks across the globe^1^. A major concern is high levels of antibiotic resistance seen in *A. baumannii*. Vaccination is one alternative to antibiotics. An effective vaccine has the potential to reduce the economic burden of bacterial infection by reducing diagnoses and treatment costs^2^. Reduced antibiotic use as a result of vaccination can also lead to reduced development of further antibiotic resistance^3^. Additionally, bacteria are less likely to develop resistance to vaccines in part because antibiotics tend to have a single mechanism of action allowing for easier mutational escape^4^. By comparison, vaccines typically work via multiple mechanisms, inducing different host responses such as specific antibody and/or T cell responses making vaccine resistance more difficult to develop. Due to the ability of *A. baumannii* to quickly and easily develop new resistance to antibiotics, an effective vaccine provides a viable alternative method of control.

One challenge for developing new vaccines against bacterial pathogens is identifying antigens that induce protective immunity. Traditional bacterial vaccines either target a secreted toxin, surface polysaccharide or contain whole, inactivated bacteria. There are potential limitations to these approaches for the development of new vaccines. Not all bacteria make a toxin or if they do, neutralization of the toxin may not protect against disease. Surface polysaccharide is poorly immunogenic on its own. Whilst this can be improved through conjugation to protein, polysaccharides are very strain specific, reducing the breadth of protection offered by the vaccine. Polysaccharide vaccines may lead to strain replacement, where infections with a different strain of the bacteria become more prevalent than those with strains in the vaccine^5^. This has been seen with the pneumococcal conjugate vaccine, the new version of which now contains 20 different polysaccharides^6^; increased from 7 in the original iteration. Whole-cell bacterial vaccines can provide a greater breadth and longevity of protection, but are also associated with increased reactogenicity; for example whole-cell pertussis was replaced with acellular pertussis in many countries^7^. Therefore, targeting a subset of bacterial proteins may be beneficial.

Down-selecting possible protein target antigens has successfully been done for the acellular pertussis and meningitis B (MenB) vaccines. For acellular pertussis, selection was based upon the predicted role of the proteins in pathogenicity and relative ease of manufacture. For the MenB vaccine, a reverse vaccinology approach was used^8^ where brute force expression of all 350 antigens identified the optimum combination; this was effective but demanding of resources. *A. baumannii* is a complex bacterial pathogen, expressing around 3836 genes^9^. Whilst many of these are intracellular and may not be accessible to antibody mediated vaccine protection, there are still many proteins encoded on the bacterial cell surface that might be potential vaccine targets; one study estimated there were at least 300 outer membrane proteins^10^. An alternative approach, called reverse vaccinology 2.0 uses the host immune response after infection to identify protective antigens^11^.

Having identified potential antigens, the method of delivering them to the host immune response can shape the strength of the protective response. Several novel vaccine platforms have emerged recently with the potential to target bacterial pathogens, including outer membrane vesicles (OMVs), produced naturally during the growth of many Gram-negative bacteria^12^. Native OMVs from the New Zealand outbreak strain of *Neisseria meningitidis* have been successfully incorporated into the Bexsero 4CMenB vaccine^13^; they may also act as adjuvants in this context. As well as native OMVs, recombinant OMVs can be used as antigen delivery systems. This can be achieved by expressing antigens on the surfaces of bacteria^14^ and engineering them to hypervesiculate by deleting components of the Tol-Pal system^15^. The GSK generalized modules for membrane antigens (GMMA) platform utilizes OMVs with a modified lipid A structure. Vaccine antigens can then also be conjugated onto the GMMA as a “plug and play” vaccine technology where different antigens can be incorporated onto the same backbone^16^.

An alternative vaccine platform is messenger RNA (mRNA). mRNA vaccines have several advantages including the speed and ease with which they can be produced and scalability^17^. The SARS-CoV-2 pandemic has shown mRNA vaccines can be highly effective^18^, particularly when they incorporate N1-methylpseudouridine to mask the RNA from the cell intrinsic immune response. An important development that contributed to the efficacy of mRNA vaccines was incorporation into a delivery vehicle to aid entry into cells and protect the mRNA from degradation by nucleases. Lipid nanoparticles (LNPs) are the most clinically advanced RNA vaccine formulation, with all SARS-CoV-2 mRNA vaccines using LNPs. LNPs typically consist of an ionizable lipid, a cholesterol variant, a helper lipid and a PEGylated lipid to encapsulate and protect the mRNA, though alternative approaches may be required for the delivery to mucosal surfaces. The use of mRNA vaccines against pathogenic bacteria has considerable potential. *Mycobacterium tuberculosis* mRNA vaccines encoding the MPT83 secreted lipoprotein^19^ and *M. leprae* protein Hsp65^20^ both provided some protection against *M. tuberculosis* challenge in mice. Additionally, using LNP formulated mRNA encoding F1 and V antigens, mice were protected against lethal *Yersinia pestis* challenge^21^. Similarly, *Listeria monocytogenes* antigen encoding mRNA formulated in LNPs provided some protection against challenge in C57BL/6J mice^22^. The modular properties of mRNA vaccines allow for quick and easy vaccine development and show potential for use against bacterial pathogens as well as viral.

Here we describe the identification of immunogenic and protective *A. baumannii* vaccine antigens using two different methods, as well as the development of *Escherichia coli* derived recombinant OMVs (rOMV) and mRNA vaccines. We compared different RNA vaccine platforms and routes of delivery. Intranasally delivered unmodified mRNA encoding OXA-23 led to rapid bacterial clearance and reduction of disease following infection.

## Results

### Use of polyclonal mouse and human sera to identify potential antigens

**Fig. 1:**
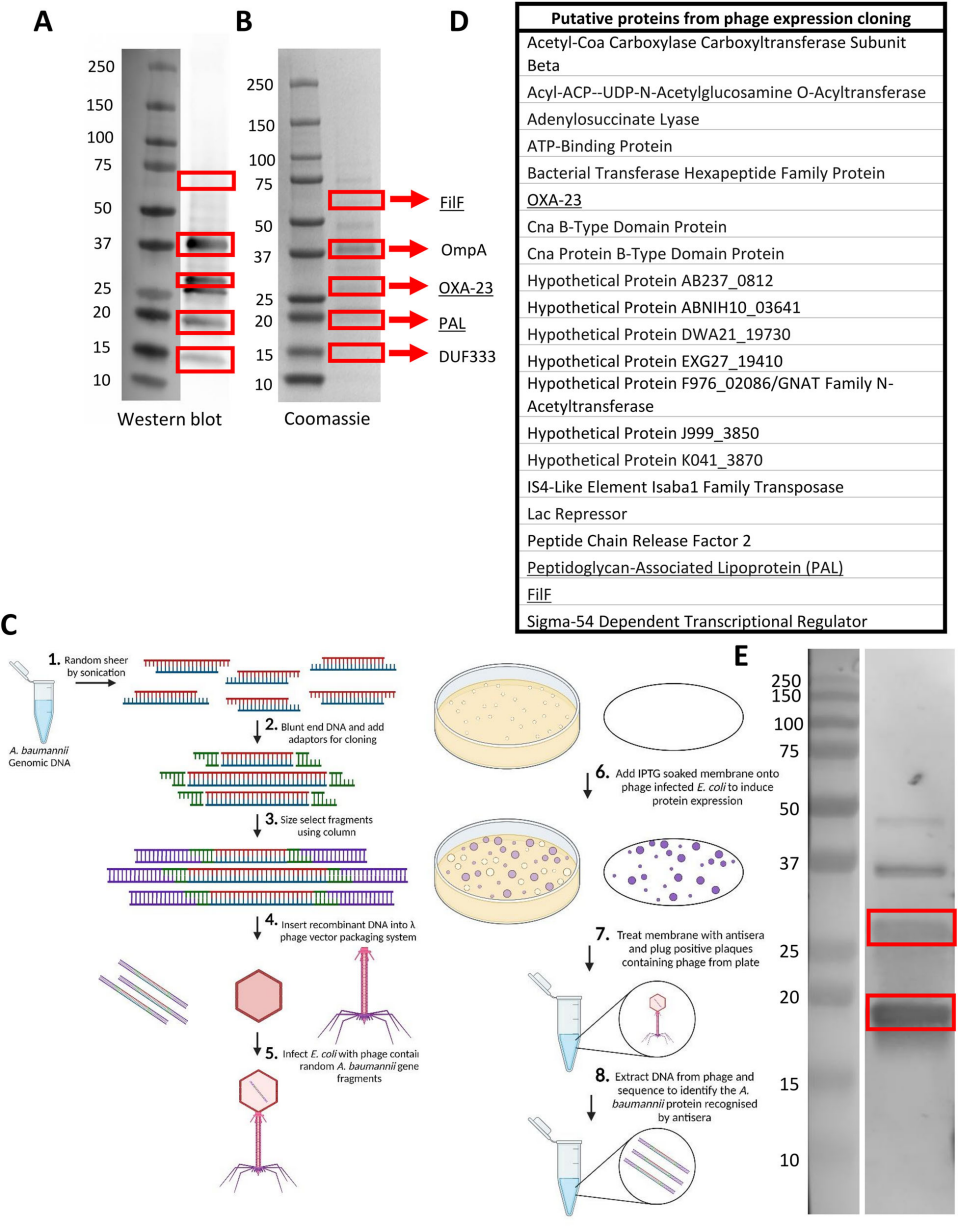
Three proteins identified via both phage expression cloning and mass spectrometry using serum from *A. baumannii* immunised mice. *A. baumannii* BAL_276 OMVs separated using SDS-PAGE to perform a Western blot (**A**) using sera from mice immunised subcutaneously with BAL_276 derived OMVs and for staining with Coomassie blue (**B**) to extract proteins for mass spectrometry analysis. Using an *A. baumannii* BAL_276 lambda phage library (**C**) and the anti-sera, phage expression cloning was used to identify putative proteins (**D**). Underlined proteins represent those identified via both methods. OMVs separated via SDS-PAGE were transferred onto membranes for western blot using convalescent patient sera (**E**). Panel (**C**) drawn with BioRender.

To identify potential vaccine antigens, sera from mice vaccinated intramuscularly with *A. baumannii* BAL_276 derived OMVs were used in two different antigen identification assays, mass spectrometry and phage expression cloning. *A. baumannii* OMVs were run on an SDS-PAGE gel and polyclonal sera from previously OMV immunised mice^23^ used in a Western blot to identify proteins recognized following immunisation (Fig. 1A). The corresponding bands of a Coomassie stained SDS-PAGE gel were extracted and analysed by mass spectrometry (Fig. 1B). This method identified five proteins: putative pilus assembly protein FilF (FilF), outer membrane protein A (OmpA), carbapenem hydrolysing class D β lactamase Oxa23 (OXA-23), peptidoglycan-associated lipoprotein (PAL) and domain of unknown function 333 (DUF333).

In parallel, phage expression cloning was performed using a lambda phage library containing random fragments of the *A. baumannii* BAL_276 genome (Fig. 1C). This library was screened using the same polyclonal mouse sera, leading to multiple positive hits. Sequencing of positive phage colonies identified 21 proteins (Fig. 1D), three of which overlapped with those identified via mass spectrometry. The three antigens recognized by antisera and identified via both methods were FilF, OXA-23 and PAL. Having identified putative antigens using mouse sera, we tested whether they were recognized by human antibodies from individuals with known history of *A. baumannii* infection. We performed a Western blot against OMV derived from strains *A. baumannii* BAL_084, BAL_191 and BAL_276 OMVs using convalescent serum from patients hospitalized with *A. baumannii* infection. There were bands of the same size as OXA-23 and PAL (Fig. 1E); with the PAL band more pronounced compared to control serum (Supplementary Fig. S1). This suggests that upon natural infection an immune response against OXA-23 and PAL is raised by the human immune system. We therefore focused on these 2 antigens.

**Fig. 2:**
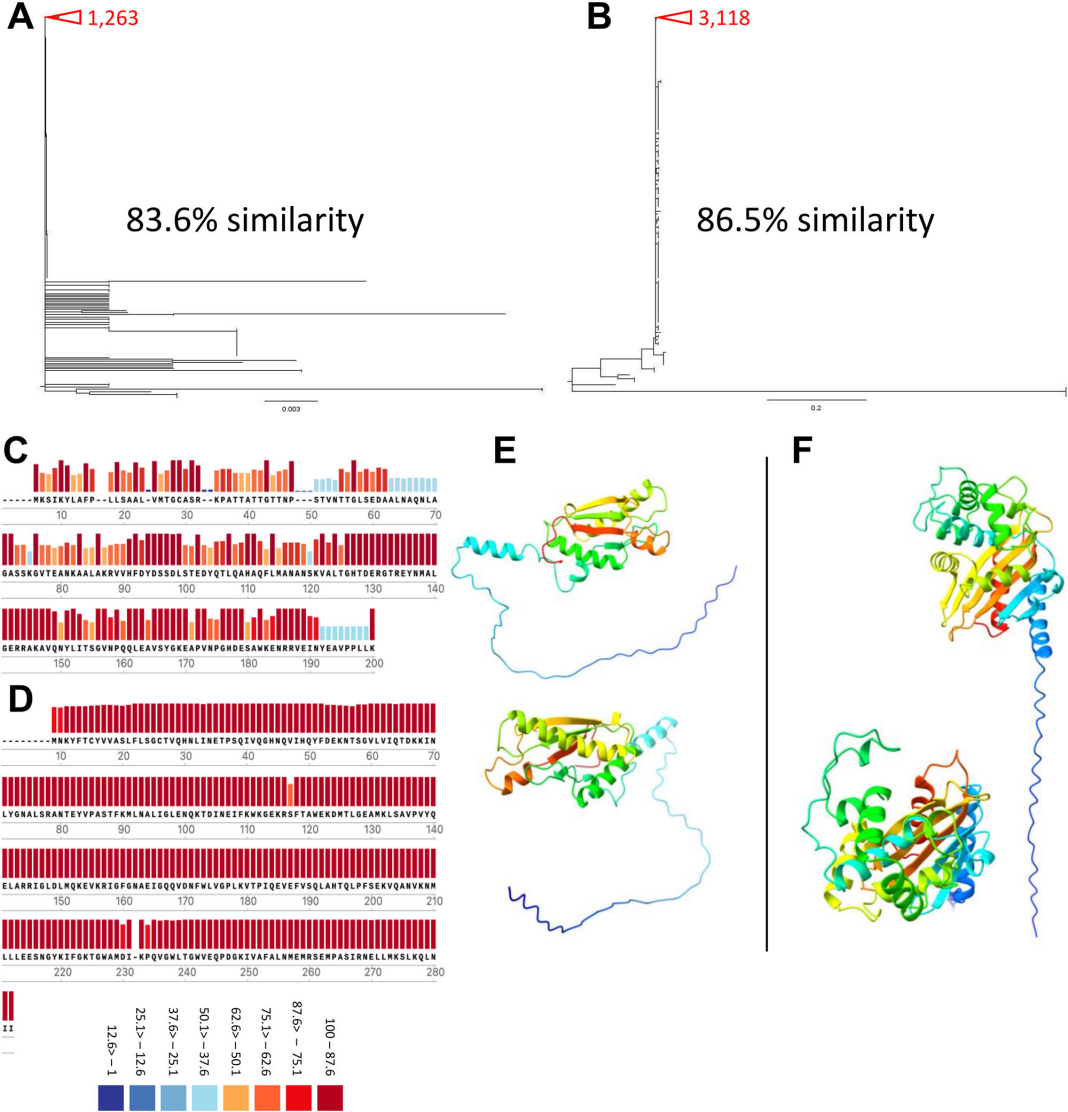
OXA-23 and PAL show high levels of sequence homology between different *A. baumannii* strains. Amino acid sequences for OXA-23 and PAL were collected from the NCBI protein database and aligned. Using MEGA-11 maximum likelihood phylogenetic trees were generated for OXA-23 (**A**) and PAL (**B**). The trees were rooted arbitrarily, and the best model was selected for each tree using MEGA-11 model selector. Due to the size of the phylogenetic trees large numbers of identical sequences were collapsed; this is depicted by red triangles with the number of collapsed sequences noted. The consensus sequence of PAL and % similarity of each amino acid (**C**); the consensus sequence of OXA-23 and % similarity of each amino acid (**D**). AlphaFold predictions of consensus sequence of PAL (**E**) and (**F**) OXA-23 consensus sequence. Both protein predictions were coloured blue to red from the N to C terminal domains; structured regions indicated by helices, unstructured regions by long chain.

To determine the level of similarity of the identified antigens between different strains of *A. baumannii*, amino acid sequences were collected from the NCBI database. The sequences were aligned, and maximum likelihood phylogenetic trees generated using MEGA11 for OXA-23 (Fig. 2A) and PAL (Fig. 2B). The longest branches containing identical sequences were collapsed, with the number of identical sequences in each collapsed branch noted next to it. PAL was the most highly conserved, with 86.5% of sequences were identical and 83.6% of the OXA-23 sequences were identical. Consensus sequences were also produced from the collected amino acid sequences for both PAL (Fig. 2C) and OXA-23 (Fig. 2D). The consensus sequence of PAL was less conserved than OXA-23. PAL had a highly conserved region between amino acids 125 and 150 whereas OXA-23 was highly conserved throughout. The consensus sequences were also used to generate an AlphaFold prediction of PAL (Fig. 2E) and OXA-23 (Fig. 2F); this indicated they both have a structured region and a long unstructured tail.

### rOMVs expressing OXA-23 and PAL were immunogenic and protected against infection

**Fig. 3:**
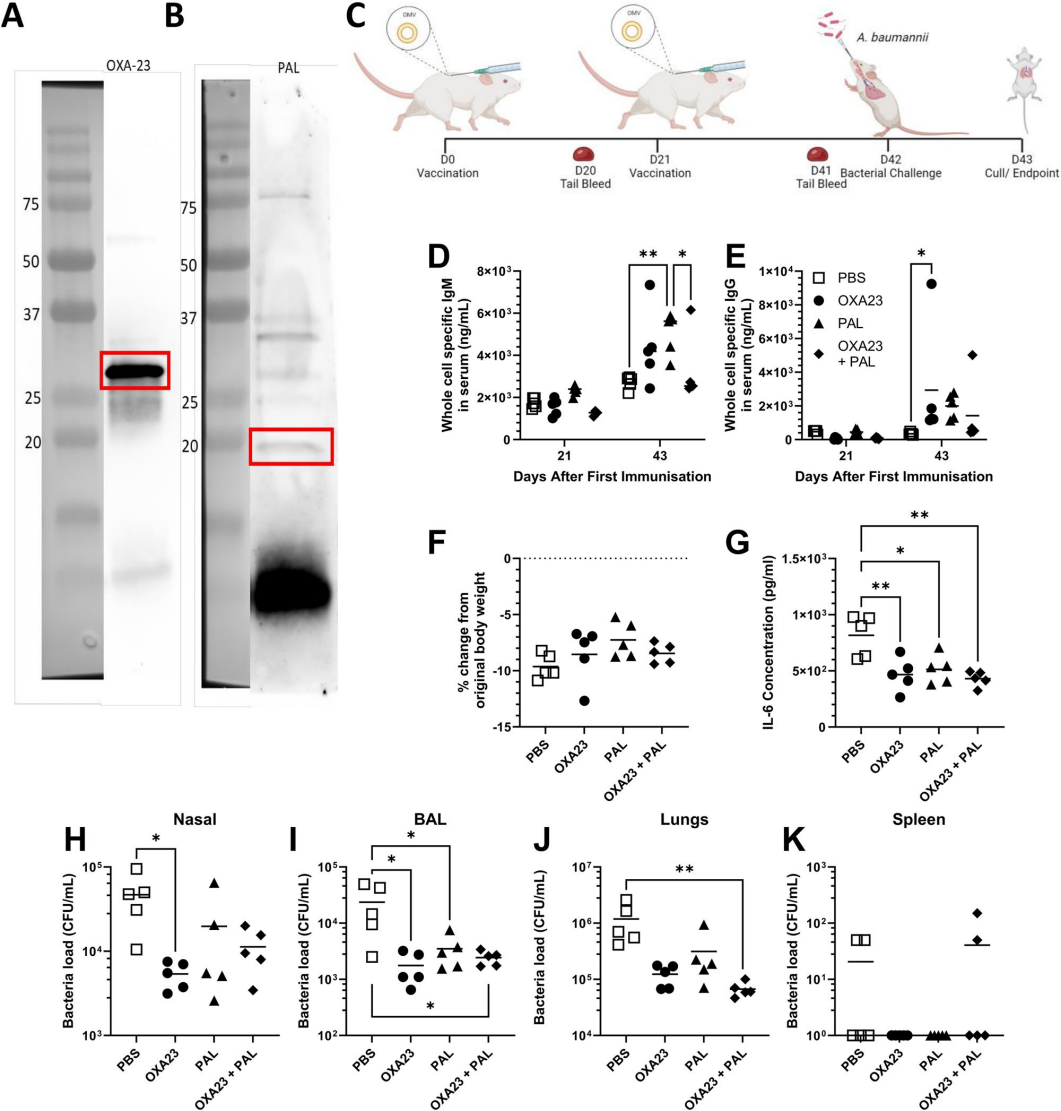
Subcutaneous vaccination with rOMVs provides protection against intranasal challenge. Δ*TolR::AmpR E. coli* possessing each plasmid to express either OXA-23 or PAL were grown in liquid culture with 400 mM IPTG to induce protein expression over 24 h. The rOMVs were separated by SDS-PAGE for Western blot using serum from mice immunised subcutaneously with BAL_276 derived OMVs to show expression of OXA-23 (**A**) and PAL (**B**) proteins on *E. coli* derived rOMVs. BALB/c mice were vaccinated subcutaneously with 500 FU (~5 μg) *E. coli* derived rOMVs expressing PAL or OXA-23 individually or in combination in a prime boost approach with 21-day intervals. Mice were challenged intranasally with *A. baumannii* BAL_276 21 days after the boost and culled 24 h later (**C**). Whole bacterial-cell specific IgM (**D**) and IgG (**E**) titres were measured by ELISA. Weight loss (**F**), IL-6 level in the lungs (**G**) and bacterial loads in nasal lavage (**H**), BAL fluid (**I**), lung homogenate (**J**) and spleen homogenate (**K**) were measured 24 h after challenge. One-way ANOVAs with Tukey tests were performed to determine significant differences between groups where ** shows *p* < 0.01, * shows *p* < 0.05; *n* = 5 per group.

Having identified two potential antigens, we tested them for immunogenicity and protection using two different vaccine platforms rOMV and mRNA. Hypervesiculating *ΔTolR*::*AmpR E. coli* can be used to produce rOMVs expressing antigens of interest on their surface. We generated *E. coli* expressing either OXA-23 or PAL and confirmed this via Western blot using polyclonal serum from mice intramuscularly immunised with *A. baumannii* BAL_276 derived OMVs. Bands were visible for both OXA-23 (Fig. 3A) at around 30 kDa and PAL (Fig. 3B) at around 19 kDa after 24-hour induction with IPTG during rOMV production.

To determine whether *E. coli* derived rOMVs could protect against intranasal challenge with *A. baumannii* BAL_276, mice were immunised subcutaneously in a prime boost regime (21 days apart) with OXA-23 rOMVs and PAL rOMVs individually or in combination (Fig. 3C). We used subcutaneous immunisation because our previous studies had indicated improved protection using this method with *A. baumannii* derived OMVs.

There was no difference in the level of whole *A. baumannii* bacterial cell specific IgM at day 21 between any of the mice (Fig. 3D). By day 43 the immunised mice had developed an *A. baumannii* whole bacterial cell specific IgM response that was significantly increased in the PAL immunised mice compared to the PBS and combined groups. The anti *A. baumannii* whole bacterial cell specific IgG response 21 days after prime was low (Fig. 3E). However, at day 43, a significantly increased IgG response began to develop in the immunised groups (*p* < 0.05 OXA-23 immunised mice compared to PBS).

Mice were intranasally challenged with *A. baumannii* BAL_276 on day 42. All mice lost weight 24 h after challenge (Fig. 3F). All immunised groups had significantly reduced lung inflammation, measured by lung supernatant IL-6 (*p* < 0.05, Fig. 3G). Significantly fewer bacteria were recovered from the nose of mice immunised with OXA-23 rOMVs compared to naïve mice (Fig. 3H, *p* < 0.05). Significantly fewer bacteria were recovered from the BAL fluid of mice immunised with OXA-23 and PAL, either individually or in combination compared to the naïve mice (Fig. 3I, *p* < 0.05). However, there was no significant difference between numbers of bacteria recovered from the nose of mice immunised with PAL, or the two antigens in combination compared to the naïve mice. In the lungs, only the mice vaccinated with both OXA-23 and PAL antigens in combination had significantly reduced bacterial recovery compared to the naïve mice (Fig. 3J, *p* < 0.01). There was very little dissemination of bacteria from the lungs to spleen in any of the groups, including the naïve mice (Fig. 3K). As a whole, the results show that *A. baumannii* antigen expressing rOMVs can induce a systemic IgG and IgM response in mice, leading to a significant reduction of bacterial load in the airways. This suggests both antigens in combination and individually induce some level of protective efficacy in an rOMV format.

### OXA-23 and PAL mRNA vaccines induce systemic IgG responses and reduce bacterial burden in the airways

**Fig. 4:**
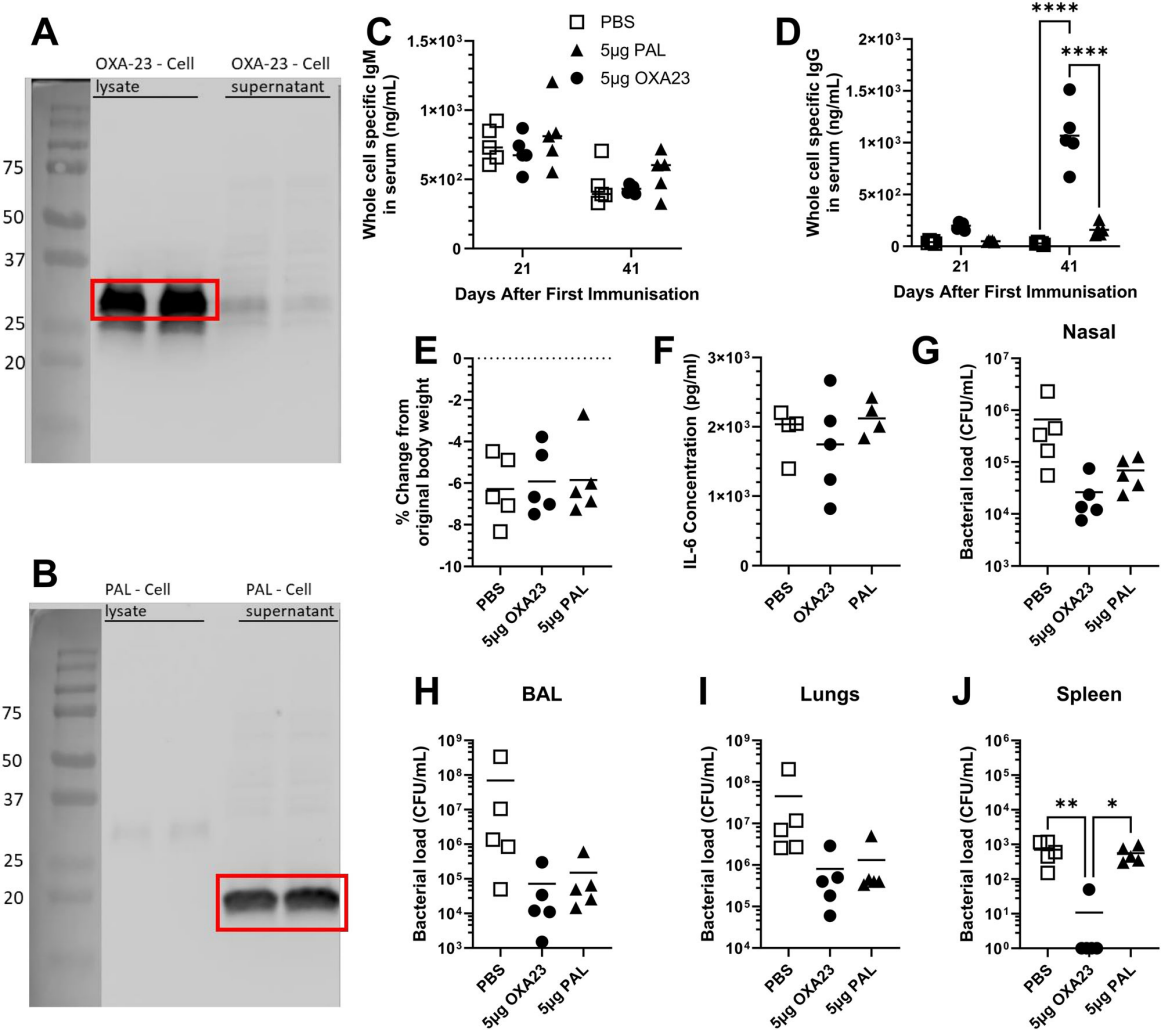
Intramuscular vaccination with OXA-23 encoding mRNA provides protection against intranasal challenge. HEK293T cells were transfected with mRNA encoding either OXA-23 (**A**) or PAL (**B**) and left for 24 h. Cell lysates and cell supernatants were separated by SDS-PAGE for Western blot with serum from mice previously immunised BAL_276 derived OMVs. BALB/c mice were vaccinated intramuscularly with 5 μg LNP formulated mRNA encoding PAL or OXA-23 in a prime boost approach with 21-day intervals. Mice were challenged intranasally with *A. baumannii* BAL_276 21 days after the boost and culled 24 h later. Their whole bacterial cell specific IgG (**C**) and IgM (**C**) titres were measured by ELISA. Weight loss (**E**), IL-6 level in the lungs (**F**) and bacterial loads in BAL fluid (**G**), nasal lavage (**H**), lung homogenate (**I**) and spleen homogenate (**J**) were measured 24 h after challenge. One-way ANOVAs with Tukey tests were performed to determine significant differences between groups where **** shows *p* < 0.0001, *** shows *p* < 0.001, ** shows *p* < 0.01, * shows *p* < 0.05; *n* = 5 per group.

We tested whether a second vaccine platform, mRNA, was able to induce a protective immune response to the same antigens. The genes *OXA-23* and *pal* were cloned into an mRNA plasmid vaccine backbone for in vitro transcription. Since bacterial proteins have different post-translational modification to eukaryotes, we wanted to confirm that antigens expressed by eukaryotic cells could be recognized by antibodies from mice exposed to native *A. baumannii* antigens. HEK293T cells were transfected with mRNA encoding the *OXA-23* and *PAL* genes. The proteins within the cell lysates and supernatants were evaluated by Western blot using serum from mice immunised intramuscularly with *A. baumannii* BAL_276 rOMVs. The antisera bound strongly to OXA-23 expressed by HEK293T cells (Fig. 4A), which was located within the cell lysate. The antisera also bound strongly to the PAL expressed by HEK293T cells (Fig. 4B), but PAL was found in the cell supernatant. Therefore, mammalian cells are capable of producing bacterial antigens OXA-23 and PAL from mRNA vaccine constructs that are recognized by sera from mice immunised with *A. baumannii*.

To determine the in vivo efficacy of the OXA-23 and PAL mRNA vaccines, the mRNA was formulated into LNPs, and mice were immunised in a prime boost regime (21 days apart) with LNP formulated mRNA vaccines individually. Neither vaccination induced a significantly greater OMV-specific IgM response than the naïve group (Fig. 4C). There was no difference in the whole-cell specific IgG detected in the serum at day 21 (Fig. 4D), however the anti-bacterial IgG titres induced by both OXA-23 and PAL increased by day 43. The amount of whole-cell specific IgG detectable in the mice immunised with OXA-23 was significantly (*p* < 0.0001) increased at day 43 compared to naïve and PAL immunised mice. Mice were then challenged on day 42 intranasally with *A. baumannii* BAL_276 and culled 24 h later. All mice lost weight (Fig. 4E), and all mice had high levels of IL-6 in the lung supernatant 24 h after challenge (Fig. 4F). Mice immunised with OXA-23 and PAL both had a reduction in bacterial burden compared to PBS treated mice, but this was not significant compared to naïve mice (Fig. 4G; *p* < 0.01). Although mice immunised with OXA-23 and PAL had reduced bacterial recovery from their BAL fluid of ~2.5 logs, this was not significantly reduced compared to naïve mice (Fig. 4H; *p* < 0.05). The same pattern was seen in the lungs (Fig. 4I; *p* < 0.05). Immunising mice with PAL had no effect on bacterial dissemination to the spleen (Fig. 4J). However, OXA-23 mRNA immunisation significantly reduced dissemination of bacteria to the spleen (*p* < 0.05) compared to either the naïve group or PAL immunised mice. Only one mouse from the OXA-23 immunised group had bacteria recoverable from the spleen compared to all 5 mice in both the PAL and naïve groups. These data suggest in an mRNA format that OXA-23 can significantly reduce bacterial burden and induce a significant systemic IgG response.

**Fig. 5:**
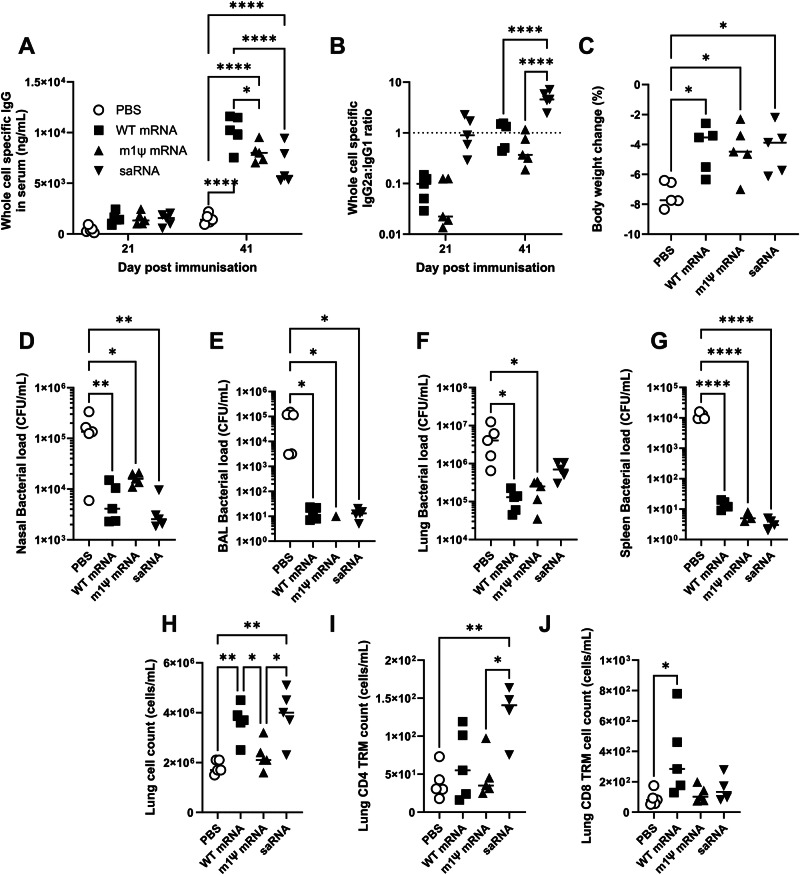
Changing RNA platform does not provide additional benefit over WT RNA. BALB/c mice were vaccinated intramuscularly with 1 μg of LNP formulated wild type mRNA or m1ψ mRNA encoding OXA-23 in a prime boost approach with 21-day intervals. Whole bacterial cell specific IgG (**A**) and the IgG2a/1 ratio (**B**) were measured in serum after prime and boost immunisation. Mice were challenged intranasally with *A. baumannii* BAL_276 21 days after the boost and culled 24 h later. Body weight change (**C**) and bacterial loads in nasal lavage (**D**), BAL fluid (**E**), lung (**F**) and spleen homogenate (**G**) were measured 24 h after challenge. Cells collected from lung were counted (**H**) and stained for CD4 Trm (**I**) and CD8 Trm (**J**). One-way ANOVAs with Tukey tests were performed to determine significant differences between groups where **** shows *p* < 0.0001, *** shows *p* < 0.001, ** shows *p* < 0.01, * shows *p* < 0.05; *n* = 5 per group.

Since it demonstrated better protection, we focussed on the OXA-23 antigen for further evaluation. For viral derived antigens, the incorporation of N1-methylpseudouridine (m1Ψ) instead of uridine can improve the level of protection for virally derived antigens^24^ as can the use of saRNA^25^, we wished to evaluate the impact of different RNA vaccine platforms to deliver OXA-23. To investigate whether the substitution of uridine (unmodified) with m1ψ (modified) would improve the protective efficacy of the vaccine, four groups of 5 mice were treated with PBS or 1 µg of either wildtype UTP (WT mRNA), m1ψ UTP (m1ψ mRNA) mRNA vaccine or self-amplifying RNA (saRNA). All vaccines led to statistically significant increases in whole bacteria specific serum IgG compared to the PBS group on both pre-boost and pre-challenge timepoints of 21- and 41-days post immunisation (Fig. 5A). There was no significant difference in the antibody concentration between the vaccines at any timepoint. However, the saRNA group had a significantly higher IgG2a: IgG1 ratio, indicating a Th1 skew (Fig. 5B; *p* < 0.01). The vaccinated groups had statistically significant protection against weight loss from infection (Fig. 5C). All mRNA vaccines significantly reduced bacterial load recovered from the nasal (Fig. 5D) and BAL lavage (Fig. 5E). Likewise, all groups had significantly reduced bacterial recovery from the lung compared to the PBS group (Fig. 5F). Both mRNA groups, but not saRNA, had lower bacterial loads in the spleen (Fig. 5G). We also assessed the impact of RNA type on T cell responses in the lung after infection. From flow cytometry analysis, the modified RNA had the lowest lung cell recovery, and WT and saRNA had more cells (Fig. 5H). The saRNA immunised group had significantly more CD4 Trm (defined as CD69^+^/CD103^+^) in the lungs (Fig. 5I) compared to PBS or m1Ψ groups; the WT mRNA had significantly more CD8 Trm (Fig. 5J).

**Fig. 6:**
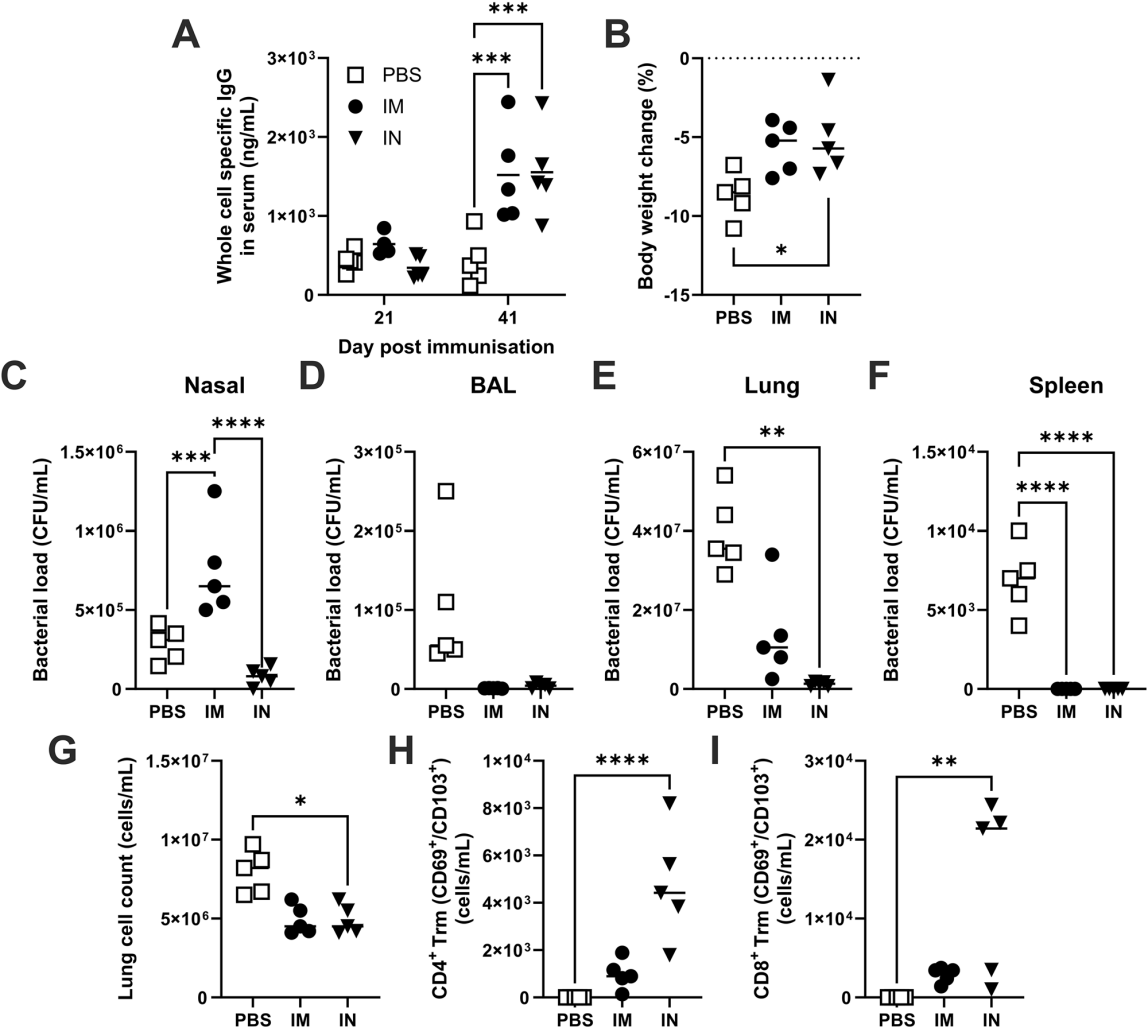
Intranasal delivered OXA-23 reduces weight loss and increases bacterial clearance. BALB/c mice were vaccinated intramuscularly (IM) or intranasally (IN) with 1 μg of wild type mRNA OXA-23 formulated with GL67 in a prime boost approach with 21-day intervals. Whole bacterial cell specific IgG (**A**) and the IgG2a/1 ratio (**B**), measured in the serum after prime or boost immunisation. Mice were challenged intranasally with *A. baumannii* BAL_276 21 days after the boost and culled 24 h later. Body weight change (**C**) and bacterial loads in nasal lavage (**D**), BAL fluid (**E**), lung (**F**) and spleen homogenate (**G**) were measured 24 h after challenge. Cells collected from lung were counted (**H**) and stained for CD4 Trm (**I**) and CD8 Trm (**J**). One-way ANOVAs with Tukey tests were performed to determine significant differences between groups where **** shows *p* ≤ 0.0001, *** shows *p* ≤ 0.001, ** shows *p* ≤ 0.01, * shows *p* ≤ 0.05; *n* = 5 per group; IN immunisation representative of 2 studies.

Having seen no advantage in m1Ψ incorporation, we investigated whether other routes of immunisation could improve protection or bacterial clearance. With an OMV based vaccine, we observed improved clearance with intranasal vaccination^23^ and hypothesised that intranasal vaccination might also improve mRNA vaccine efficacy. For intranasal delivery, we switched to a formulation based on the cationic lipid Genzyme lipid (GL) 67A, which has previously been used to deliver DNA for gene therapy^26^. Mice were immunised with 1 μg WT mRNA formulated in GL67A either intramuscularly or intranasally. Both vaccines led to statistically significant increases in whole bacteria specific serum IgG compared to the PBS group on both pre-boost and pre-challenge timepoints of 21- and 41-days post immunisation (Fig. 6A). The IM RNA group had a significantly higher IgG2a: IgG1 ratio, indicating a Th1 skew (Fig. 6B; *p* < *0.01*). There was no significant difference in the antibody concentration between the vaccines at any timepoint; we were unable to detect antigen specific IgA in the BAL or nasal lavage. Mice were then intranasally challenged with *A. baumannii*; the intranasal immunised group lost significantly less weight than the PBS group (Fig. 6C). Intranasal immunisation protected against bacterial infection, with low levels of bacterial recovery from the upper (Fig. 6D) or lower airways (Fig. 6E). There was also a significant reduction in bacterial numbers recovered from the lungs (Fig. 6F) or spleen (Fig. 6G) of the IN immunised group. IN immunisation led to a significant greater clearance of bacteria from the nose than IM immunisation. We also assessed the impact of RNA type on T cell responses in the lung after infection. IN immunisation was associated with fewer total cells in the lung following infection (Fig. 6H), but an increase in both CD4 (Fig. 6I) and CD8 (Fig. 6J) Trm. We, therefore, conclude it is possible to deliver mRNA vaccines IN and they offer improved protection.

## Discussion

The aim of the study was to identify and test protective antigens against *A. baumannii*. Two key antigens were identified through phage expression cloning and mass spectrometry, OXA-23 and PAL. High levels of similarity across the majority of sequences collected from the NCBI database suggests these antigens should be broadly protective. These antigens were carried forward to develop novel rOMV and mRNA vaccines against *A. baumannii*. We showed that when tested as rOMV and mRNA vaccines OXA-23 and PAL were immunogenic in BALB/c mice, and provided protection against a carbapenem resistant *A. baumannii* clinical strain^27^. We then improved the protection provided by mRNA encoded OXA-23 by delivering it intranasally.

OXA-23 is a carbapenem-hydrolysing class D β-lactamase found globally^28–30^. To our knowledge OXA-23 has not previously been shown to be a protective vaccine antigen for *A. baumannii*; though a previous study has demonstrated that serum from people infected with *A baumannii* can recognize OXA-23 as one of several antigens in a larger screen^31^. Bioinformatic analysis and reverse vaccinology have both shown β-lactamases to have good potential for use as vaccines against *A. baumannii*^32,33^. Our study uses 2 other methods of antigen isolation and then experimental challenge models to cross validate the informatic approaches previously published. OXA-23 has been shown to interact with the outer membrane porins OmpA and CarO at the periplasmic surface^34^. If this is the case, perhaps there are other interactions between OXA-23 and other outer membrane proteins that allow OXA-23 to associate with the outer membrane but also render OXA-23 accessible to antibodies. Our recent work has shown OXA-23 to be membrane bound and targetable by antibodies^35^. With this being the case, it is likely that antibodies can work via multiple mechanisms not only through neutralization of the lactamase activity of the enzymes but also aiding in opsonophagocytosis. Perhaps in a case such as *A. baumannii*, an OXA-23 vaccine could help to neutralize OXA-23 carbapenemase and increase sensitivity to some antibiotics as seen previously when serum anti-OMV antibodies improved *A. baumannii* sensitivity to quinolone based antibiotics in a serum bactericidal assay^36^.

PAL has been previously identified and highlighted as a potential vaccine antigen for *A. baumannii* by reverse vaccinology^37^, bioinformatic approaches^38^ and immunoproteomic approaches^39^. PAL knockout strains of *A. baumannii* are more susceptible to killing by detergent and have increased sensitivity in serum bactericidal assays. Recombinant PAL vaccines have previously been shown to protect 40% of mice against lethal intranasal *A. baumannii* challenge^39^; as have DNA vaccines^40^. Our own data have shown PAL to be effective at reducing inflammation in the lungs and reducing bacterial load in the respiratory tract when administered as an rOMV vaccine. This agrees with a previous study where PAL induced strong IgG antibody responses and a mixed Th1, Th2 and Th17 response when used as a vaccine antigen^40^. How PAL and OXA-23 compare to other antigens that have been proposed for *A. baumannii* needs further evaluation. At least 20 different antigens have been tested across a range of platforms, routes and mixed with different adjuvants^41^, but as yet none has entered clinical trials.

Expressing antigens of interest on the surface of rOMVs has been shown to elicit more robust immune responses against the antigen than adjuvanted recombinant protein alone. Mice generated significantly increased antibody titres to rOMVs expressing Omp22 from *A. baumannii* on their surface compared to alum adjuvanted, recombinant Omp22^42^. Additionally, rOMVs can be used to induce an immune response against multiple pathogens. *Salmonella* Typhimurium rOMVs engineered to express pneumococcal surface adhesin A significantly enhanced pneumococcal antibody responses in mice compared to a recombinant pneumococcal surface adhesin A vaccine, while also inducing a strong *S*. Typhimurium antibody response^43^. OMV vaccines likely elicit enhanced immunity due to their size, facilitating entry into lymph nodes; antigen-presentation by APCs after being phagocytosed; carriage of pathogen-associated molecular patterns acting as adjuvants and presentation of antigens in their native conformation within a bacterial membrane^12^. More exact immunoprotective mechanisms vary between OMVs however. *Salmonella* OMVs promote strong T and B cell responses, induce the production of TNF, IL-12 and expression of CD86 and MHC II molecules on dendritic cells^44^. Comparatively, *Pseudomonas aeruginosa* OMVs induced the secretion of IL-8 in the lungs^45^ and *Neisseria meningitidis* OMVs aid neutrophil recruitment, inducing TNF-α and IL-1β production and upregulating CXCL8, CCL3 and CCL4^46^. We observed that rOMVs elicit robust immune responses in mice, resulting in a reduction of inflammation in the lungs and reduced bacterial burden upon infection but determining the exact mechanism of action requires further work.

Despite previous suggestions that RNA-based bacterial vaccines resulted in inferior protection against bacterial infection, here we showed that mRNA vaccines encoding *A. baumannii* antigens were able to induce whole-cell specific IgG responses in mice and reduce bacterial burden in the lungs, respiratory tract and reduce dissemination to the spleen. The difference in vaccine efficacy between RNA vaccines encoding OXA-23 and PAL could be due to PAL being secreted from mammalian cells, subjecting PAL to post-translational modifications in the secretory pathway, which have been reported previously to dampen immune responses to nucleic acid vaccines^47^. During mammalian protein expression the addition of N-linked sugars onto bacterial proteins can alter their epitopes and affect antigen presentation to MHC II molecules^48^. Modifying nucleosides can dampen the innate immune response to sequence and secondary structures formed by mRNA thereby improving protein translation efficiency and antibody recognition^49^. However, in the case of OXA-23, we saw no benefit of including m1Ψ in the RNA. This may reflect the need for T cells in protection against *A. baumannii*; we^24^ and others^50^ have previously observed that RNA modification reduces the induction of T cells. We also explored whether intranasal delivery of RNA could improve responses, this has been seen to be effective for the model antigen OVA^51^ and viral antigens^52^; but this is the first-time intranasal delivery of mRNA encoded bacterial antigens has been used.

Further issues encountered during this program of work include variability in bacterial challenge doses and determining how many bacteria enter the lungs. To account for this, large volumes of challenge dose (100 μl) were used to ensure bacteria enter the lungs and to try and reduce variability. We also could not ensure proper folding of bacterial antigens in mammalian cells; however, we were able to see binding of mammalian expressed protein in western blot using OMV immunised mouse anti-serum. Furthermore, determining precise antigen concentration on the rOMVs is challenging. OMVs were quantified by Lowry assay, and it was determined that approximately equivalent doses of 5 μg of total protein were used to immunize mice; however, we do not know the percentage make up of vaccine antigen vs *E. coli* antigen. Further work could be performed to elucidate the immunological mechanisms behind each vaccine type. However, this was beyond the scope of the work presented here and will be performed in the future.

Here we demonstrate the protective efficacy of a novel vaccine antigen for *A. baumannii*, OXA-23 and show its efficacy in two vaccine types, *E. coli* derived rOMV and mRNA-LNP based vaccines. This is the first time an mRNA-LNP vaccine has been used for *A. baumannii* and the first intranasal delivery of a bacterial antigen, reducing bacterial burden during in vivo infection. In addition to helping to prevent infection, it could also lead to improved treatment outcomes and work synergistically with antibiotic therapies to better treat *A. baumannii* infection. A major consideration is how to deploy a vaccine for *A. baumannii* which is relatively rare, often only occurring as a nosocomial consequence of hospitalization; a possible approach is to include in a combination vaccine with broad spectrum protection targeting syndromes rather than specific pathogens^53^.

## Methods

### Native OMV production

Clinical isolates of *A. baumannii* strains BAL_276 was isolated from ICU patients in Ho Chi Minh^27^. All *A. baumannii* strains were grown at 37 °C, 200 rpm in Lysogeny broth (LB) or statically at 37 °C on HiCrome Acinetobacter Agar Base (HIMEDIA M1938: Trafalgar). OMVs were generated as described^23^. Briefly, bacteria were grown planktonically in LB overnight. The culture was centrifuged at 7000 × *g* for 15 min at 4 °C and the supernatant containing the OMVs was vacuum filtered using a 0.45μm filter. The filtered supernatant was then ultracentrifuged at 100,000 × *g* for 2 h at 4 °C to pellet the OMVs. OMVs were quantified by incubation with lipophilic dye FM4-64 in a black 96 well polypropylene microtiter plate. Fluorescence was then read using an EnVision Plate Reader (Perkin Elmer) with excitation 520 nm and emission 700 nm and recorded as fluorescent units (FU).

### OMV immunisation of mice to generate polyclonal sera

All animal studies were performed in accordance with the United Kingdom’s Home Office guidelines under animal study protocol number (P4EE85DED). All work was approved by the Animal Welfare and Ethical Review board at Imperial College London. Studies also followed NC3Rs guidelines, and all work was carried out in biosafety level-two facilities.

The studies performed here used specific pathogen free, female, 6–8-week-old BALB/c mice (weight between 20 and 25 g), bought from Charles River Laboratories (Hertford, UK). Mice were maintained in individually ventilated cages, housed in groups of five with water and food provided ad libitum. The cages were kept in a specific pathogen free room at 20–24 °C with 55 ± 10% humidity. Animal group sizes presented in figure legends.

For euthanasia, conscious mice were injected with 200 μl Pentoject (Animalcare) i.p.; this was followed with a secondary confirmation of death. This allows for an intact trachea for bronchoalveolar lavage (BAL).

To generate polyclonal sera, mice were intramuscularly with 100 fluorescent units (FU) of OMVs from *A. baumannii* (from strain BAL_276) in a 50 μl volume as described^35^. Mice were immunised in a prime-boost regime with a 3-week interval between immunisations. Serum was collected 3 weeks after boost.

### SDS-PAGE, Western blotting and mass spectrometry

For western blot, 500 FU of OMV (~5 µg) were run down mini-PROTEAN TGX gels and the proteins were transferred onto PVDF membranes using Trans-Blot Turbo Mini PDVF Transfer Packs and a Trans-Blot Turbo. Membranes were blocked with 1% bovine serum albumin in phosphate buffered saline, washed with 0.1% PBS-tween then coated with serum from mice intramuscularly immunised with OMVs (1:200 dilution).

Anti-mouse IgG-HRP (IgG-HRP Bio-Rad STAR120P, 1:1000) was used for detection of OMV-specific IgG and blots were visualized using western HRP chemiluminescent substrate (Fisher Scientific WBLUR) and imaged using a Celvin S Chemiluminescent imager (Biostep).

For identification by mass spectrometry, 500 FU OMV were run on a separate mini-PROTEAN TGX gel in parallel to the Western blot gel. The gel was stained with InstantBlue Coomassie protein stain and imaged. The protein bands corresponding to the Western blot were excised and an in-gel tryptic digestion kit (Thermo Scientific 89871) was used to extract the protein ready for analysis by mass spectrometry.

The digested samples were analysed by online nano-LC-ESI-MS and MS/MS using a Synapt G2-S (Waters), quadrupole time-of-flight mass spectrometer. Online analysis was performed using a nano-Acquity Ultra Performance LC (Waters). A BEH C_18_ column (75 μm inner diameter ×15 cm length) was used for all analyses. Samples were eluted from the column using 0.1% formic acid in water and a 10–45% acetonitrile gradient over 45 min. For data-independent acquisition, LC-MS^E^ was performed, with fragmentation achieved using CAD with argon as the collision gas.

Protein identification – Raw LC-MS and MS/MS data were processed with ProteinLynx Global SERVER version 3.01 (Waters) to identify proteins, searching against UniProt specific databases (UniProt Consortium, 2021).

### Phage expression cloning

Phage expression cloning was performed using the methods presented by Lodes et. al.^54^ and using the Agilent Lambda ZAP II Undigested Vector Kit (23 6201-12). In brief (Fig. 1C), the lambda phage library was generated by extracting genomic DNA from *A. baumannii* BAL_276 and sheering via sonication. The ends were blunted with DNA ligase and dNTPs before adding on *Eco*RI adaptors for cloning. Finally, fragments were selected using DNA size selection columns and ligated into the lambda ZAP II expression vector pBluescript SK. The phage library was titrated to estimate plaque forming units per ml and the quality of the library was checked by colony PCR and amplified DNA fragments were analysed via gel electrophoresis to confirm a variation in the size of gDNA fragments. After confirming the phage library was of good quality, the library was amplified, and aliquots were stored at −80 °C for future use.

Negative phage containing empty vector was also produced to be used as negative pick phage. This allowed for sera from mice, immunised intramuscularly with BAL_276 derived OMVs, to be pre-adsorbed with *Escherichia coli* proteins and remove any antibodies from the sera binding to *E. coli* prior to screening against the *A. baumannii* phage library.

To perform the primary screen, *E. coli* XL1-Blue MRF cells were grown overnight in LB broth at 30 °C, shaking at 200 rpm. The following morning the culture was centrifuged, and the bacterial pellet was resuspended in 10 mM MgSO_4_ and diluted to an OD_600_ of 0.5 in 10 mM MgSO_4_. In a microcentrifuge tube, the *E. coli* XL1-Blue MRF cells were mixed with the positive phage and incubated at 37 °C for 15 min, to allow the phage to attach to and begin to infect the *E. coli* XL1-Blue MRF cells, prior to mixing the cells and phage in NZY top agar and pouring the mixture on top of NZY agar plates. After the top agar had set, the plates were incubated at 42 °C until plaques began to form. Nitrocellulose membranes that had been pre-soaked in 10 mM IPTG were placed on top of the plates and the plates were incubated at 37 °C overnight. The following morning, the plates were cooled at 4 °C for 15 min before removing the membranes, which were then washed in 0.1% PBS-Tween20 and then blocked using 1% bovine serum albumin in 1% PBST with rocking.

The membranes were washed again before adding the pre-adsorbed mouse serum to each membrane and incubating at room temperature with rocking. The membranes were washed again prior to the addition of AP conjugated anti-mouse IgG to membranes and incubation at room temperature with rocking. Finally, membranes were washed before adding NBT/BCIP until purple spots developed. Positive plaques corresponding to the purple spots on the membrane were removed (plugged phage) and placed into SM buffer containing chloroform and stored at 4 °C. To reduce potential contamination, a secondary screen was performed. At the initial step of mixing the *E. coli* XL1-Blue cells and the phage the plugged phage from the primary screen was used instead of the phage from the phage library.

To excise the phagemids, plugged phage from the secondary screen were vortexed and incubated at room temperature before storing at 4 °C. *E. coli* XL1-Blue MRF and SOLR cells were grown overnight in LB broth with supplements, centrifuged and resuspended to an OD_600_ of 1.0 in 10 mM MgSO_4_. The *E. coli* XL1-Blue MRF cells, plugged positive phage and ExAssist helper phage were mixed and incubated at 37 °C. LB broth with supplements was then added to the tubes and the tubes were incubated at 37 °C with shaking for 2–3 h. The lambda phage particles were then lysed by incubating the tubes for 20 min at 65–70 °C and the cell debris was pelleted at 1000 × *g* for 15 min. The supernatant was added to a microcentrifuge tube containing SOLR cells and incubated for 15 min at 37 °C before plating the mixture onto LB ampicillin plates and incubating overnight at 37 °C. Individual colonies were picked into LB broth containing ampicillin to grow overnight and extract plasmids to be sequenced. The sequencing data was then blasted against the *A. baumannii* genome to identify the proteins.

### Human sera

Serum samples from the ‘Microbial Products and effects on the host’ project obtained at the point of admission were linked to anonymized patient data provided by an NHS clinician including the date the sample was taken in relation to the point of admission. (West London REC reference 06/Q0406/20). Control human sera was collected from an anonymised donor from the CHLM-02 trial^55^.

### Phylogenetic tree analysis and AlphaFold visualisation

To produce phylogenetic trees, amino acid sequences were collected from the NCBI protein database for *bla*_*OXA-23*_ and *pal* for *A. baumannii*. Any duplicate sequences were removed and following the methods published by Hall^56^, the sequences were aligned using MEGA11 and muscle alignment with standard settings. For each protein, the model selector was used to determine the best model to use when generating the maximum likelihood tree. Each collection of aligned amino acid sequences was then used to generate phylogenetic tree files in MEGA11 using the JTT model for Oxa-23 and JTT + G for PAL. Finally, the trees were formatted in FigTree to collapse long branches containing identical sequences. Consensus sequences were generated using SnapGene. AlphaFold visualisations were generated using ColabFold^57^.

### rOMV production

To produce recombinant OMVs the pDSG254 vector was digested with *Not*I and the *bla*_*OXA-23*_ and *pal* genes were amplified from *A. baumannii* BAL_276 gDNA using the following primers:

*bla*_*OXA-23*_-Forward: GCTAGGCCCAGCCGGCCATGAATAAATATTTTACTTGCTATGTGGTTGC *bla*_*OXA-23*_-Reverse: GTCAGCGGCCGCTTAAATAATATTCAGCTGTTTTAATGATTTCATCAATAATTC

*pal*-Forward: GCTAGGCCCAGCCGGCCATGAAATCAATTAAATATTTGGCCTTTCCTC *pal*-Reverse: GTCAGCGGCCGCTTATTTTAATAGAGGAGGAACCGCTTC

Hypervesiculating *TolR*::*AmpR E. coli* were made electrocompetent and transformed via electroporation with the pDSG254 vectors containing the *bla*_*OXA-23*_ and *pal* genes. The transformed *E. coli* were grown in LB broth with ampicillin and chloramphenicol at 37 °C with shaking until they reached OD_600_ 0.5–0.6 and IPTG was added to a concentration of 400 μM to induce protein expression and the tubes were incubated overnight at 37 °C overnight.

To separate the rOMVs, the bacterial culture was centrifuged at 7200 × *g* and the supernatant was removed and filtered using a 0.45 μm Stericup. The filtered supernatant was then ultracentrifuged at 100,000 × *g* to pellet the rOMVs and the rOMVs were resuspended in PBS. To quantify the rOMVs, they were incubated at room temperature with FM4-64 lipophilic dye. The fluorescence was read using a FLUOstar Omega with excitation 520 nm, emission 700 nm. The data was expressed as fluorescence units (FU) relative to the volume of sample added to the well.

### mRNA production and formulation

To produce mRNA *bla*_*OXA-23*_ and *pal* were amplified out of *A. baumannii* BAL_276 gDNA using the following primers:

*bla*_*OXA-23*_-Forward: GCTAGCTCTTCCATGAATAAATATTTTACTTGCTATGTGGTTGC *bla*_*OXA-23*_-Reverse: GCTAGCTCTTCGTTAAATAATATTCAGCTGTTTTAATGATTTCATCAATAATTC

*pal*-Forward: GCTAGCTCTTCCATGAAATCAATTAAATATTTGGCCTTTCCTC *pal*-Reverse: GCTAGCTCTTCGTTATTTTAATAGAGGAGGAACCGCTTC

The genes were cloned into the LG005 vector, an mRNA vaccine plasmid with a T7 promoter (Φ6.5), clean cap AG initiation site, alpha globin 5’UTR, Kozak sequence, beta globin 3’UTR and a segmented poly-A tail consisting of 60As-G-60As^58^. Alternatively, they were cloned into a VEEV saRNA backbone.

Amplified genes were digested with *Sap*I, and the genes were ligated into the vector. The vector was linearised with *BamH*I and *Nsi*I before blunting with T4 DNA polymerase and dNTPs (NEB) following manufactures instructions to remove the 3’ overhang and reduce dsRNA formation. The mRNA was in vitro transcribed using the HiScribe T7 High Yield RNA Synthesis Kit (NEB E2040S) with clean cap AG (TriLink) followed by LiCl purification. In one study uridine was replaced with N1-methylpseudouridine.

Using an N/P ratio of 6, the mRNA was formulated in lipid nanoparticles using the lipid components C12-200, DSPC, cholesterol and DMPE-PEG2000, in a PBS 10% sucrose solution. Using the RiboGreen RNA quantitation assay kit (Life Technologies, UK) the amount of mRNA present in the LNP formulations was quantified both with and without 2% Triton X-100 to determine the concentration of mRNA in the formulation and % of encapsulation compared to an RNA standard. Additionally, the zeta potential and polydispersity index were determined using the ZetaSizer Ultra (Malvern Instruments), folded capillary cuvettes were used to make the measurements. The mean diameters and polydispersity were determined using dynamic light scattering.

For generation of RNA/GL67A complexes, lipid and RNA were complexed using a 3:1 molar charge ration of nucleic acids to lipid as previously described^59^.

### In vitro expression

1.5 × 10^6^ HEK293T cells were seeded into 6 well plates and transfected with 1 ug per well mRNA encoding either OXA-23 or PAL using Lipofectamine MessengerMAX (Invitrogen) and Opti-MEM (ThermoFisher) and left for 24 h. Cells were lysed and lysates and cell supernatants were separated by SDS-PAGE for Western blot with serum from mice previously immunised BAL_276 derived OMVs.

### In vivo vaccination and challenge

Mice were vaccinated in a prime boost approach with 21-day intervals. For rOMVs, mice were given 500 FU subcutaneously. Where mice were immunised with a combination of the two antigens, 250 FU each antigen were administered subcutaneously. For the initial mRNA study comparing antigens, mice were immunised with 5 μg mRNA formulated in LNP. For subsequent studies comparing RNA platforms, mice were immunised with 1 μg LNP formulated mRNA or saRNA. For study comparing IN and IM, mice were immunised with 1 μg GL67A formulated mRNA. IM vaccination was in a 50 μl volume, IN vaccination in a 100 μl volume.

Mice were challenged intranasally 21 days after being boosted with *A. baumannii* BAL_276 and culled 24 h after bacterial challenge. For intranasal challenge mice were anaesthetized via inhalation of isoflurane before being intranasally challenged with 5 × 10^7^ CFU per mouse in 100 μl sterile PBS. Mice were weighed prior to challenge and then again 24 h later before being culled.

To determine bacterial loads in the bronchoalveolar lavage and nasal lavage fluid the samples were serially diluted 1:10 and plated onto HiChrome Acinetobacter Agar Base plates, which were incubated overnight at 37 °C to determine the colony forming units per ml. For the lungs and spleens, both lobes of the lungs and the whole spleens were mashed using the gentleMACS tissue dissociator. The lung and spleen homogenates were then serially diluted 1:10 and plated onto HiChrome Acinetobacter Agar Base plates and incubated overnight at 37 °C.

### Bacterial specific ELISA

ELISAs were performed on *A. baumannii* BAL_276 whole bacterial cells. Bacteria were cultured until they reached OD_600_ 1.0 and pelleted via centrifugation to remove the LB broth and resuspend in carbonate coating buffer to OD_600_ 0.5. Bacterial suspension was added to sample wells and anti-κ anti-λ antibodies were used to coat the standard curve wells. After washing with 0.05% PBST and blocking with 1% BSA in PBS, plates were coated with mouse serum on the sample wells or isotype control IgG/IgM on the standard wells. Anti-mouse IgG HRP or anti mouse IgM HRP were used to detect total IgG and IgM and TMB substrate was used to develop the ELISA, using 2 N H_2_SO_4_ to stop the reaction before reading the plates at 450 nm using a FLUOstar omega microplate reader. In some studies, OMV was used as the coating antigen.

### Cytokine ELISA

To measure IL-6 levels in the BAL fluid and lungs, the R&D systems mouse IL-6 DuoSet ELISA kit was used (DY406; LOD 15.6 pg/ml).

### Statistical analysis

Calculations as described in figure legends (One-way ANOVAs with Tukey tests were performed to determine significant differences between groups) were performed using GraphPad Prism 9 (GraphPad Software Inc., La Jolla, CA, USA).

## Supplementary information

Supplementary information

Raw Data

## Data Availability

All data generated or analysed during this study are included in this published article [and its supplementary information files].
