# Supplementary material for: Intranasal delivery of mRNA expressing newly identified Acinetobacter baumannii antigens protects against bacterial lung disease
Source: NPJ Vaccines. 2025 Jul 4;10:144. doi: 10.1038/s41541-025-01202-0 (PMC12227653; doi:10.1038/s41541-025-01202-0)
Supplement: Supplementary file 1 — Supplementary information [file 41541_2025_1202_MOESM1_ESM.pdf]

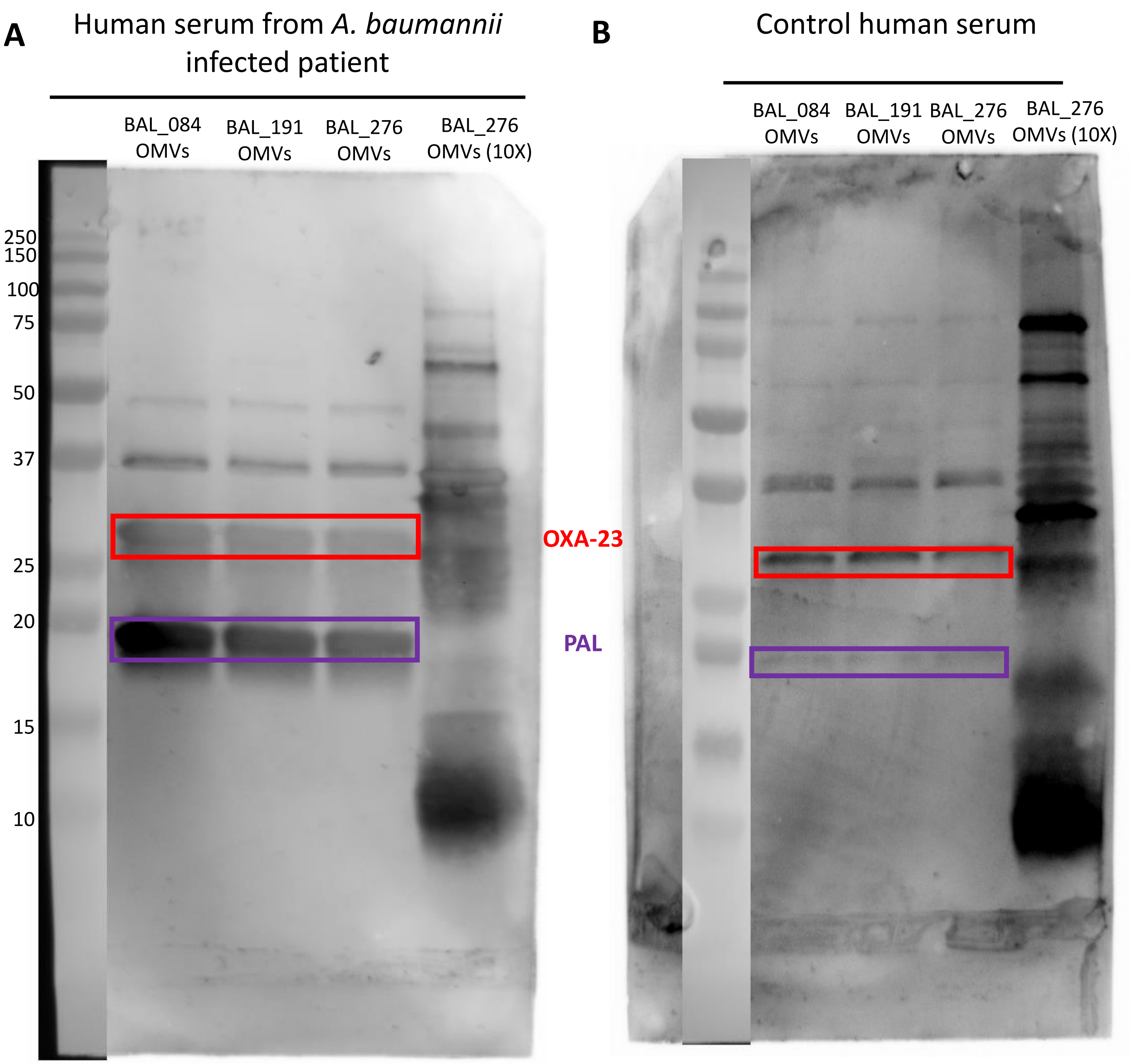


**Figure S1 | After infection with *A. baumannii* antibody responses are raised against OXA-23 and PAL in humans.** Different capsule type and GC2 subclade clinical isolate derived OMVs were separated via SDS-PAGE and the proteins were transferred onto membranes for western blot using human serum from an infected patient **(A – 1E)** and uninfected control patient **(B)**. The bands where OXA-23 and PAL were visible were highlighted in red. The western image capture system automatically overlays the ladder.

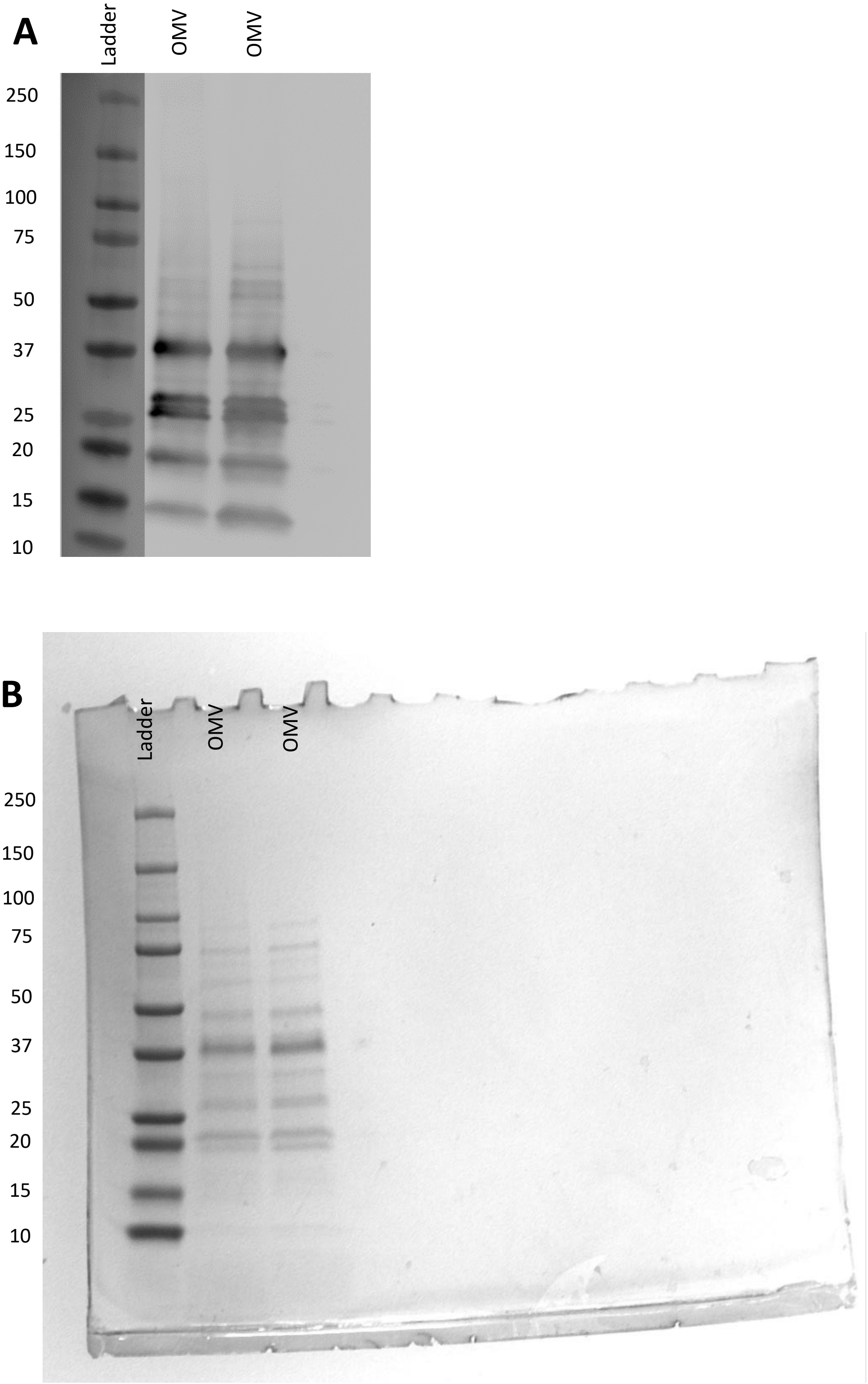


**Figure S2: Panels from Figure 1, full blots.** *A. baumannii* BAL_276 OMVs separated using SDS-PAGE to perform a western blot (A – 1A) using sera from mice immunised subcutaneously with BAL_276 derived OMVs and for staining with Coomassie blue (B – 1B) to extract proteins for mass spectrometry analysis. (Cropped blots in Figure 1)

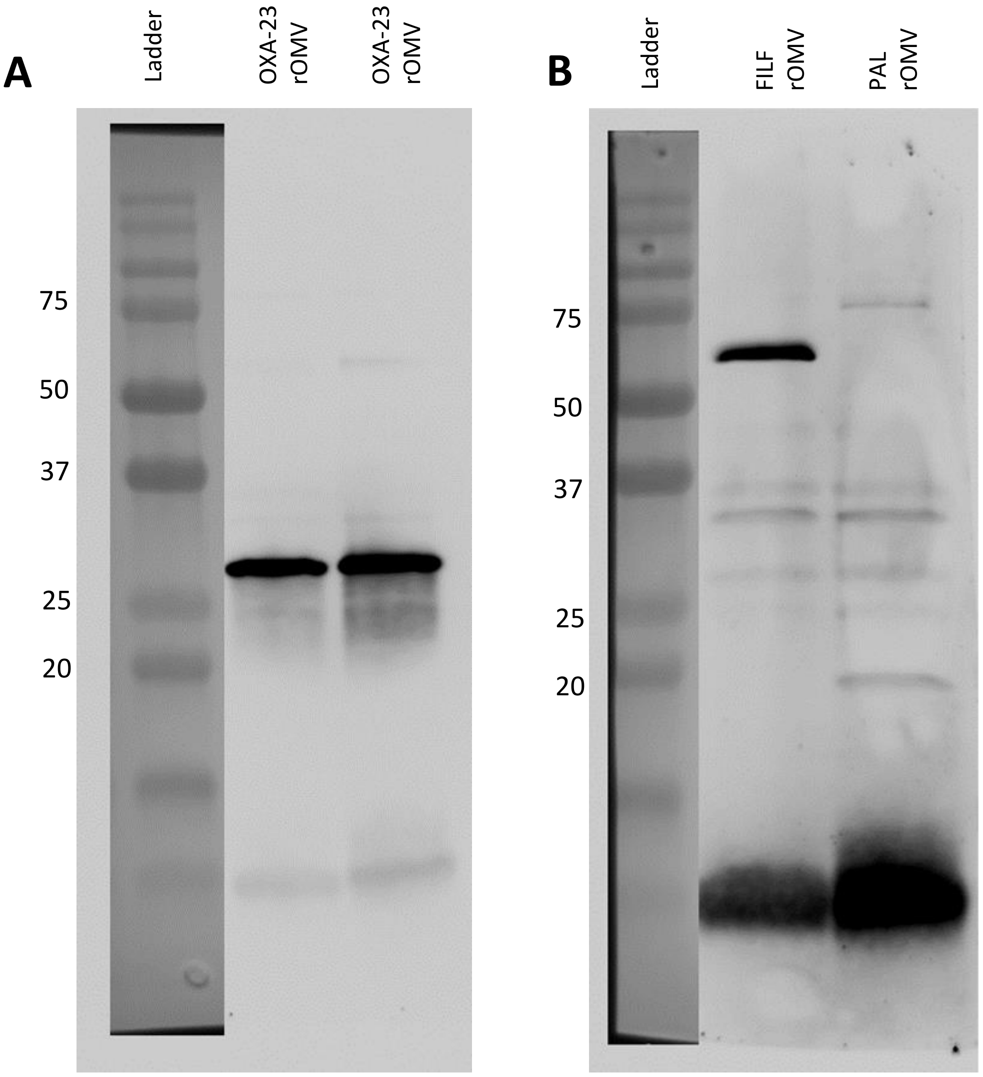


**Figure S3: Blots from Figure 3.** Δ*TolR::AmpR E. coli* possessing each plasmid to express either OXA-23 or PAL were grown in liquid culture with 400mM IPTG to induce protein expression over 24 hours. The rOMVs were separated by SDS-PAGE for western blot using serum from mice immunised subcutaneously with BAL_276 derived OMVs to show expression of OXA-23 **(A – 3A)** and PAL **(B – 3B)** proteins on *E. coli* derived rOMVs.

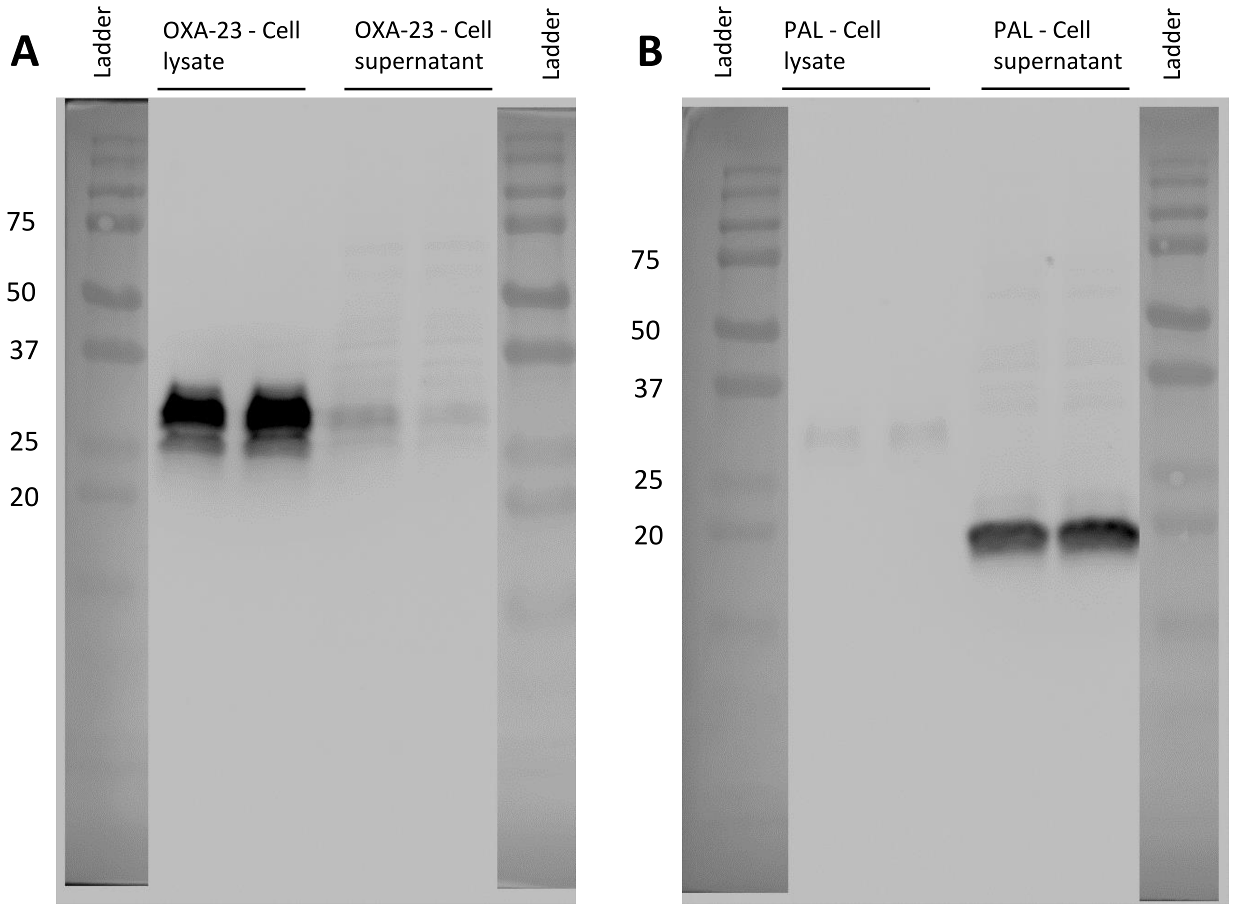


**Figure S4: Blots from Figure 4**. HEK293T cells were transfected with mRNA encoding either OXA-23 **(A – 4A)** or PAL **(B – 4B)** and left for 24 hours. Cell lysates and cell supernatants were separated by SDS-PAGE for western blot with serum from mice previously immunised BAL_276 derived OMVs.
